# Epidemiology of drowning in children rescued by Shiraz EMS from 2020 to 2022

**Published:** 2023-12

**Authors:** Mohammad Javad Moradian, Behnaz Rastegarfar, Farahnaz Fooladband

**Affiliations:** ^*a* Department of Health in Emergencies and Disasters, Faculty of Management and Medical Informatics, Shiraz University of Medical Sciences, Shiraz, Iran. ^; ^*b* Department of Disaster and Emergency Health, Faculty of Public Health, Tehran University of Medical Sciences, Tehran, Iran. ^; ^*c* Disaster and Emergency Medical Management Center, Shiraz University of Medical Sciences, Shiraz, Iran. ^

## Abstract

**Background:**

Drowning is the third cause of death among children aged 1 to 5 years worldwide, which makes it a serious issue requiring much attention and awareness in parents and caregivers. Most cases of drowning are initially reported to pre-hospital emergency services. By studying the data and identifying patterns, decision-makers can implement targeted interventions, raise awareness, and allocate resources effectively to address this critical problem. This study aimed to determine the epidemiology of drowning in children rescued by Shiraz EMS from 2020 to 2022.

**Methods:**

This cross-sectional study was conducted on 37 children reporting drowning incidents in Shiraz from 2020 to 2022. For data collection, we used a checklist that included variables such as sex, age, address, place of drowning, time, and the evaluation result by the EMS team, including trauma with drowning. The data were analyzed using SPSS software for statistical analysis (Version 23).

**Results:**

Drowning incidents predominantly occur among children above the age of 5 (65%), boys (73%), in summer (59%), and in areas near roadways (62%). The majority of drowning incidents occurred in pools (54%), rivers (24%), and ponds and wells (14%). Out of the total cases, 24% resulted in fatalities, 57% were transferred to the hospital, and 19% received outpatient treatment. CPR was performed for 43% of the cases, with a success rate of 44%. Additionally, 52% of them experienced trauma simultaneously with drowning.

**Conclusions:**

The findings of this study highlight the specific age groups, gender, and locations where these incidents are more prevalent. Policymakers, healthcare professionals, and communities can use this information to develop targeted interventions to educate parents, caregivers, and the community about the importance of water safety measures, supervision, and CPR training. Further studies are needed about child drowning in pre-hospital emergency centers in Iran, with a larger sample size that aligns with similar cultural, geographical, and social contexts.

**Keywords:**

Epidemiology, Drowning, EMS

